# The Role of c-Jun and Autocrine Signaling Loops in the Control of Repair Schwann Cells and Regeneration

**DOI:** 10.3389/fncel.2021.820216

**Published:** 2022-02-09

**Authors:** Kristjan R. Jessen, Rhona Mirsky

**Affiliations:** Department of Cell and Developmental Biology, University College London, London, United Kingdom

**Keywords:** PNS, repair Schwann cell, c-Jun, regeneration, nerve injury, Schwann cell

## Abstract

After nerve injury, both Schwann cells and neurons switch to pro-regenerative states. For Schwann cells, this involves reprogramming of myelin and Remak cells to repair Schwann cells that provide the signals and mechanisms needed for the survival of injured neurons, myelin clearance, axonal regeneration and target reinnervation. Because functional repair cells are essential for regeneration, it is unfortunate that their phenotype is not robust. Repair cell activation falters as animals get older and the repair phenotype fades during chronic denervation. These malfunctions are important reasons for the poor outcomes after nerve damage in humans. This review will discuss injury-induced Schwann cell reprogramming and the concept of the repair Schwann cell, and consider the molecular control of these cells with emphasis on c-Jun. This transcription factor is required for the generation of functional repair cells, and failure of c-Jun expression is implicated in repair cell failures in older animals and during chronic denervation. Elevating c-Jun expression in repair cells promotes regeneration, showing in principle that targeting repair cells is an effective way of improving nerve repair. In this context, we will outline the emerging evidence that repair cells are sustained by autocrine signaling loops, attractive targets for interventions aimed at promoting regeneration.

## Introduction

**Figure 1:**
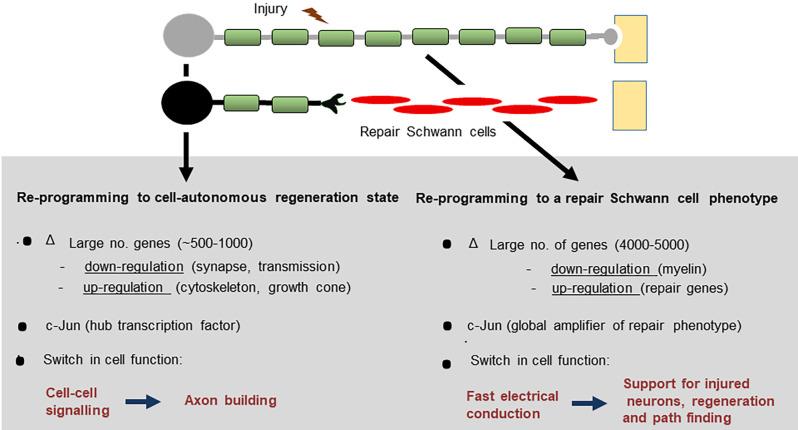
After peripheral nerve injury, both Schwann cells and neurons change function and switch to pro-regenerative states.

After nerve injury, both neurons and Schwann cells undergo radical change as they adopt phenotypes dedicated to support repair (Figure 1). The neurons reprogramme to cell-autonomous regenerative units, a process that entails a substantial change in gene expression and a switch of function from that of cell-cell signaling to that of axon building. The injury response of Schwann cells is equally impressive, involving the reprogramming of myelin and Remak cells to repair Schwann cells, a Schwann cell phenotype specialized for promoting axonal regeneration. As a result, the PNS has a striking regenerative potential (Boyd and Gordon, 2003; Jessen and Mirsky, 2005; Allodi et al., 2012; Scheib and Höke, 2013; Doron-Mandel et al., 2015; Jessen et al., 2015; Fawcett and Verhaagen, 2018; Jessen and Arthur-Farraj, 2019; Jessen and Mirsky, 2019b; Stierli et al., 2019; Nocera and Jacob, 2020; Min et al., 2021). Despite this, nerve injuries remain an important clinical problem. Workers in the field of nerve repair will be familiar with two central issues: First, that the regenerative capacity of nerves declines dramatically with age and, second, that after injury, the chronically denervated distal nerve stump gradually loses the ability to support axonal regeneration, an important reason for poor outcomes after all but the most distal nerve injuries (reviewed in Vaughan, 1992; Verdú et al., 2000; Höke, 2006; Sulaiman and Gordon, 2009; Painter, 2017).

What would cause the inherent regenerative capacity of the PNS to be compromised in this way*?* Are these two phenomena mechanistically unrelated, or perhaps linked? Might it even be possible to find common molecular mechanisms that connect these two apparently dissimilar situations in which regeneration falters?

The answers can be traced back to the identification of the transcription factor c-Jun in Schwann cells in the 1990s and to the seminal work of J. Z. Young and his colleagues half a century earlier. Not only were they the first to experimentally compare regeneration in young and aging animals (Gutmann et al., 1942), but they also provided the first evidence that axons regenerated poorly through chronically denervated distal nerve stumps (Holmes and Young, 1942). A striking elevation of c-Jun in Schwann cells after nerve injury was first reported by De Felipe and Hunt (1994), Stewart (1995), and Shy et al. (1996). The key function of this protein in Schwann cells was unknown until it was found that nerve regeneration was severely compromised in mice in which c-Jun had been selectively inactivated in Schwann cells (Arthur-Farraj et al., 2012; Fontana et al., 2012). High c-Jun levels were shown to be required for successful Schwann cell reprogramming to repair cells after injury, and for the maintenance of the repair phenotype (reviewed in Jessen and Mirsky, 2016, 2019b; Jessen and Arthur-Farraj, 2019). Most recently, defective c-Jun expression has been linked to the twin problems outlined above, namely the adverse effects of advancing age and chronic denervation on nerve regeneration (Wagstaff et al., 2021). As a necessary amplifier of the repair Schwann cell phenotype, c-Jun is therefore central both to the success and failure of nerve repair.

This review will discuss injury-induced Schwann cell reprogramming and the concept of the repair Schwann cell. We will consider the molecular control of these cells with emphasis on the transcription factor c-Jun and examine the important role of c-Jun in the regeneration deficits imposed by age and chronic denervation. Lastly, we will outline the emerging evidence that repair cells are sustained by a set of autocrine signaling loops, attractive targets for interventions aimed at promoting regeneration.

## Nerve Injury Triggers Schwann Cell c-Jun Expression

The first report of *in vivo* accumulation of c-Jun protein in Schwann cell nuclei after nerve transection in the adult was made by De Felipe and Hunt (1994), although c-Jun expression had previously been shown in cultures of perinatal Schwann cells (Monuki et al., 1989). The observation of De Felipe and Hunt was confirmed by Stewart (1995) and Shy et al. (1996) who also showed elevation of c-Jun mRNA after injury. If regeneration is prevented after nerve transection, c-Jun protein levels in the distal stump continue to rise for 1–2 weeks, to reach 80–100-fold those seen in intact nerves (Fazal et al., 2017; Wagstaff et al., 2021). In humans also, c-Jun in Schwann cells is elevated both after acute nerve injury, and in various pathological conditions (Hutton et al., 2011; Wilcox et al., 2020, 2021).

In uninjured adult nerves, c-Jun protein and mRNA are detectable, although the levels are very low compared to the injured state. Using sensitive immunohistochemistry, nuclear c-Jun can be shown in 20–30% of myelin Schwann cells of uninjured nerves, while these low basal c-Jun levels are even more easily detectable in Remak cells (Hantke et al., 2014; Klein et al., 2014).

In two situations, repair cells fail to achieve or maintain high c-Jun levels after injury, namely, as animals get older and in chronically denervated distal stumps.

Aging results in altered expression of a significant number of genes, both in uninjured and injured nerves (Painter et al., 2014; Wagstaff et al., 2021). c-Jun is among these age-sensitive genes, and c-Jun mRNA levels are significantly lower in injured nerves of aging compared to young mice. In agreement, four days after transection, c-Jun protein levels in the distal nerve stump of middle-aged mice (8–10 months) are ~50% lower than in young (1–2 months) mice (Wagstaff et al., 2021).

In small animals like rodents, nerves regenerate within weeks after an injury such as nerve crush, and c-Jun is gradually down-regulated as axons direct repair cells back to myelin and Remak cells. In the much larger human nerves, however, it is notable that repair cells distal to regenerating axons may be without axonal contact for months, as axons make their way along the nerve more proximally. This long-term, or chronic, denervation can be modeled in experimental animals by preventing regeneration of axons into the distal stump following nerve transection. Measurements of c-Jun in such chronically denervated mouse distal stumps show that denervated Schwann cells fail to maintain the high c-Jun levels they achieved 1–2 weeks after injury. Instead, c-Jun levels gradually decline, so that eight to 10 weeks after injury, c-Jun is only present at 40–50% of peak levels (Fazal et al., 2017; Wilcox et al., 2020; Wagstaff et al., 2021).

Because high c-Jun levels are instrumental in the reprogramming of myelin and Remak cells to the repair phenotype, the reduced c-Jun expression in repair cells of older animals and in chronically denervated repair cells has important implications for regeneration capacity. These issues are discussed further in subsequent sections.

While high c-Jun levels, such as those seen after injury, are associated with Schwann cell reprogramming, myelin and Remak cells tolerate a lower but significant c-Jun elevation without significant change in their phenotypes. The evidence comes from studies on c-Jun mutants and mouse models of demyelinating disease as discussed in a subsequent section (Hantke et al., 2014; Klein et al., 2014; Fazal et al., 2017).

During development, c-Jun is clearly detectable in the immature Schwann cells of perinatal nerves but decreases to low adult levels during the first 1–2 weeks after birth (Parkinson et al., 2008). It is interesting that this perinatal c-Jun expression appears not to be of great functional significance, because in mice with conditional c-Jun inactivation in Schwann cells (c-Jun cKO mice), nerve development remains essentially normal (Arthur-Farraj et al., 2012). This contrasts with the fundamental importance of c-Jun expression in Schwann cells of injured nerves.

Early observations on the mechanisms that regulate c-Jun levels showed that c-Jun is suppressed by cAMP (Monuki et al., 1989; De Felipe and Hunt, 1994), a signal that drives myelin differentiation *in vitro* and *in vivo* (Morgan et al., 1991; Bacallao and Monje, 2015; reviewed in Monk et al., 2015). On the other hand, c-Jun is elevated by Ca++, a signal implicated in activation of the Schwann cell injury response (Smith et al., 1985; De Felipe and Hunt, 1994). In recent work, several other pathways have been implicated in the regulation of Schwann cell c-Jun (reviewed in Boerboom et al., 2017; Jessen and Arthur-Farraj, 2019). More remains to be learned about how Schwann cells first detect axonal injury, what triggers increased c-Jun expression, and how Schwann cell c-Jun levels are regulated subsequently.

## When Regeneration Fails: Aging and Chronic Denervation

Numerous groups have followed up the pilot observations of Gutmann et al. (1942) and studied what happens to nerve regeneration during aging (reviewed in Vaughan, 1992; Verdú et al., 2000; Painter, 2017). This has established unambiguously that aging results in a substantial reduction in regenerative capacity, and that many of the adverse effects of aging are already seen in middle-age. Age-dependent decline in repair affects humans as well as experimental animals. Aging is accompanied by slower and less extensive axonal regrowth, delayed target innervation, and slow breakdown of myelin and axonal debris. There are significant changes in gene expression in peripheral nerves between young and aging animals, both before and after injury, including reduced expression of certain trophic factors such as GDNF and betacellulin (Painter et al., 2014; Wagstaff et al., 2021). Notably, age-dependent failures of regeneration are mainly caused by deterioration of repair Schwann cells rather than neurons (Painter et al., 2014).

While the adverse consequences of chronic denervation are particularly significant in larger animals such as humans, much has been learned about the underlying mechanisms from animal models. An early study (Holmes and Young, 1942) used rabbits to show that nerve stumps denervated for 3 months had reduced capacity to support axonal regeneration. Several groups confirmed these experiments using rats, although the time course of decline differs somewhat between reports. While regeneration support is little changed by 1 month of denervation, some studies already report deterioration by 2 months. Further reduction in regeneration is seen after 3 and 6 months of denervation (Fu and Gordon, 1995; Vuorinen et al., 1995; Sulaiman and Gordon, 2000; Jonsson et al., 2013; Ronchi et al., 2017; reviewed in Vaughan, 1992; Verdú et al., 2000). Our studies on mice show that by 2-and a-half month, the ability of chronically denervated distal stumps to support axon growth is reduced by about 50% (Wagstaff et al., 2021).

Holmes and Young (1942) concluded that “some factor is operating in these degenerated stumps to reduce the rate of advance of regenerating fibers”. It turns out the “factor” is more likely to be the absence of factors since during chronic denervation repair Schwann cells gradually decrease expression of several genes implicated in the support of injured neurons and axon growth. This includes GDNF, BDNF, NT3, and NGF (Eggers et al., 2010; reviewed in Boyd and Gordon, 2003; Höke and Brushart, 2010). Thus, the repair phenotype represents a transient differentiation state that fades with time. In larger nerves, such as those in humans, this becomes a significant obstacle to effective repair. Eventually, Schwann cell numbers also decline during chronic denervation, although this has not been extensively quantified. For several reasons, this is unlikely to explain the decline in regeneration support provided by distal stumps after 2 to 3 months of denervation (for discussion see Jessen and Mirsky, 2019b) although it will become important at later times. Thus, repair cell deterioration during chronic denervation likely proceeds in two steps: First, the dedifferentiation of repair cells, namely the gradual loss of the repair-supportive features that characterize these cells soon after injury. Second, the eventual death of dedifferentiated repair cells.

As described in a previous section, c-Jun is among the repair genes that show decreased activation after injury in middle-aged animals compared to young ones, and both in rodents and humans c-Jun fails to be maintained at high levels during chronic denervation (Wilcox et al., 2020, 2021; Wagstaff et al., 2021). To appreciate the significance of reduced c-Jun in these situations, it is helpful to analyze the Schwann cell injury response and outline the function of c-Jun in repair Schwann cells.

## c-Jun Is A Global Amplifier of The Schwann Cell Injury Response

**Figure 2:**
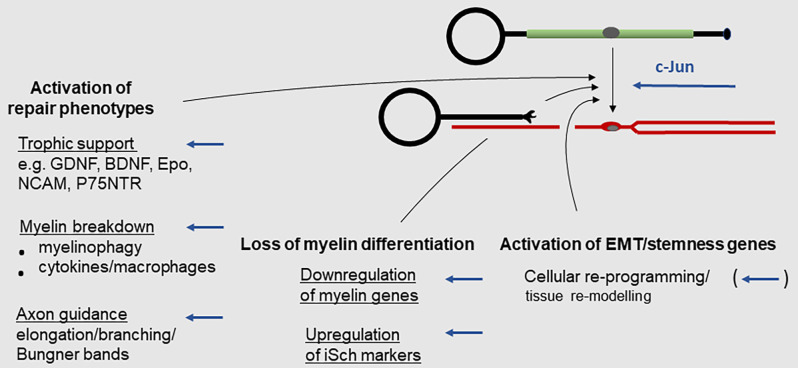
The Schwann cell injury response. Key events during the reprogramming of myelin cells to repair Schwann cells. Arrows indicate events subject to c-Jun regulation (for references see text).

**Figure 3:**
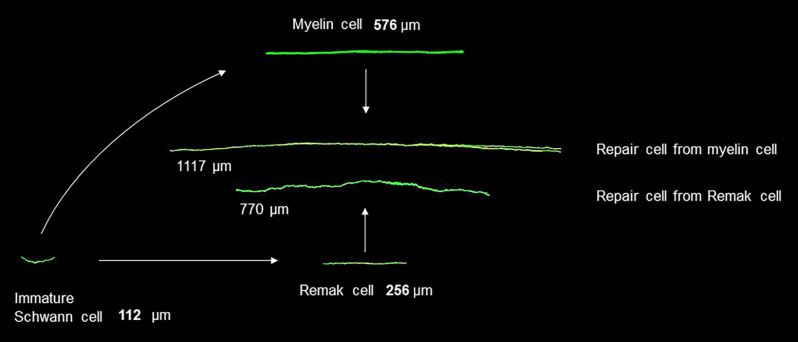
Changes in Schwann cell size during development and after injury. Shown in green are fluorescent images of immature Schwann cells, myelin and Remak cells, and the repair cells derived from them. The cells are shown to scale and illustrate cells of average length. The images are obtained after genetic labeling *in vivo* (modified from Gomez-Sanchez et al., 2017).

**Figure 4:**
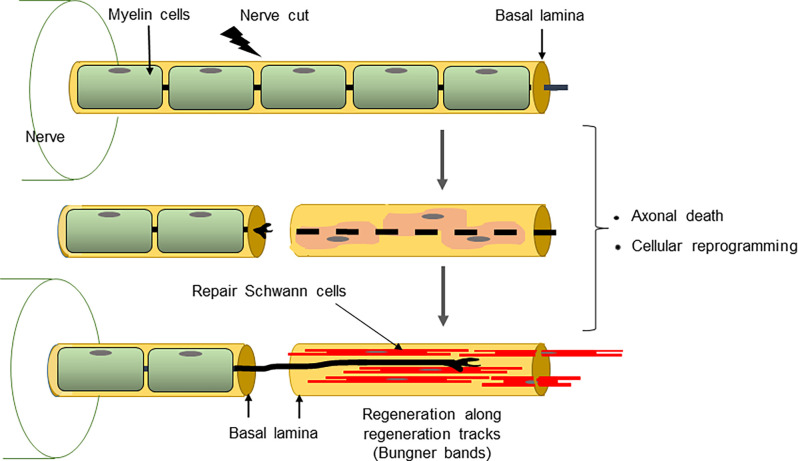
The conversion of myelin cells to repair cells and the formation of Bungner bands.

The Schwann cell injury response is more radical and better characterized in myelin cells than in Remak cells (Figures 2–4; reviewed in Chen et al., 2007; Glenn and Talbot, 2013; Jessen et al., 2015; Jessen and Mirsky, 2016, 2019b; Jessen and Arthur-Farraj, 2019; Zigmond and Echevarria, 2019; Kolter et al., 2020; Nocera and Jacob, 2020; Arthur-Farraj and Coleman, 2021; Min et al., 2021). This reprogramming event involves three types of change: (i) Up-regulation of repair phenotypes. This includes activation of trophic support for neurons and of the innate-immune response and recruitment of macrophages, up-regulation of myelinophagy for myelin clearance, and cellular elongation and branching leading to the formation of regeneration tracks (Bungner bands). (ii) Down-regulation of myelin genes; (iii) Activation of EMT/stemness genes.

As with injured neurons, the injury-induced reprogramming of myelin to repair cells is accompanied by a change in function, in this case from that of promoting fast axonal conduction to that of supporting injured neurons. And, interestingly, not only in Schwann cells but also in neurons, c-Jun is necessary for the regenerative response to injury but unimportant for development. Thus, in mice without c-Jun in neurons, axonal growth and the general development of the nervous system is essentially normal although the intrinsic ability of adult neurons in these mice to regenerate axons after an injury is compromised (Raivich et al., 2004; Zhou et al., 2004).

Among the first reported functions of c-Jun in Schwann cells was the suppression of myelination and myelin genes, rather than the promotion of nerve repair (Parkinson et al., 2004). c-Jun showed a cross-inhibitory relationship with the pro-myelin transcription factor Krox20/Egr2 and suppressed the ability of Krox20/Egr2 to activate myelin genes (Parkinson et al., 2004, 2008). Several other factors expressed in perinatal nerves share this ability to suppress myelin genes and myelination. These negative regulators of myelination include, in addition to c-Jun, Notch, Sox2, and Id2 (Parkinson et al., 2008; Woodhoo et al., 2009; Roberts et al., 2017; Florio et al., 2018; reviewed in Jessen and Mirsky, 2008). During development, negative regulation of myelination is not an important function of c-Jun, since developing and adult nerves are essentially normal in c-Jun cKO mice. During the Schwann cell injury response, however, c-Jun-mediated suppression of myelin genes has a significant role as outlined below.

**Figure 5:**
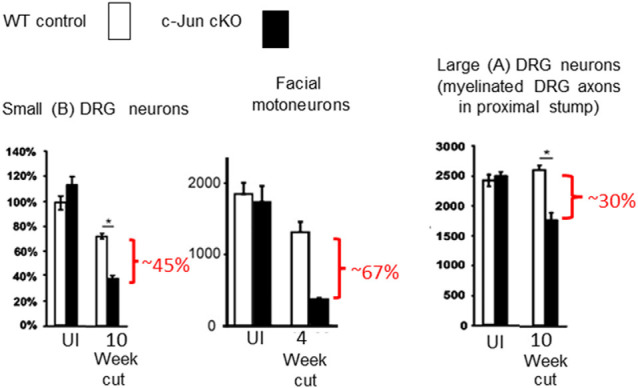
Death of injured neurons without Schwann cell c-Jun. In the absence c-Jun activation in Schwann cells, nerve transection causes extensive death of large and small DRG neurons and facial motor neurons (modified from Arthur-Farraj et al., 2012).

**Figure 6:**
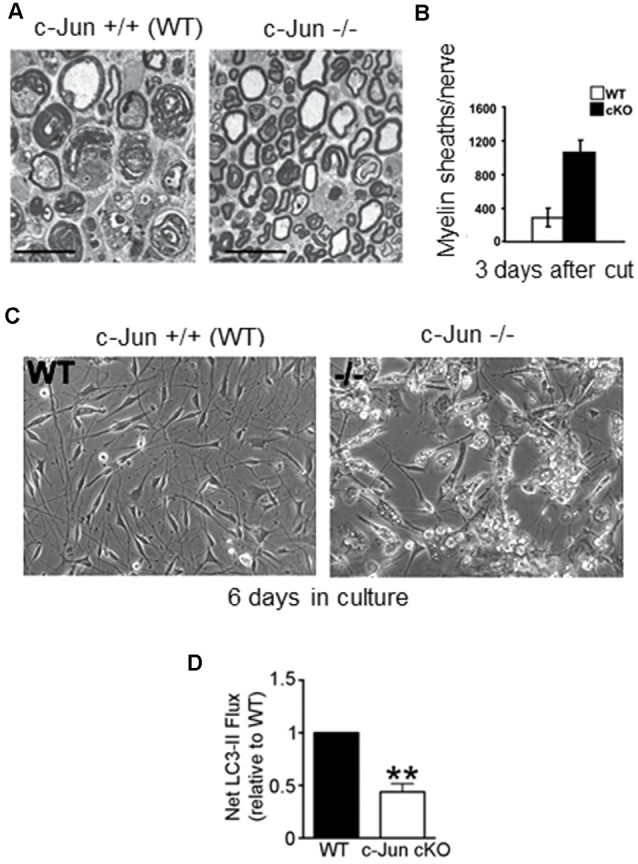
Myelin clearance and autophagy are regulated by c-Jun. **(A)** Electron micrograph of 3-day cut WT and c-Jun cKO sciatic nerves, showing relative preservation of intact myelin sheaths in the mutant; quantification shown in **(B)**. **(C)** Failure of myelin breakdown in c-Jun cKO Schwann cells in culture, shown by the persistence of myelin debris in mutant cells maintained for 6 days *in vitro*. **(D)** c-Jun cKO nerves show reduced autophagic flux (LC3 ll accumulation) compared to WT nerves (modified from Gomez-Sanchez et al., 2015).

**Figure 7:**
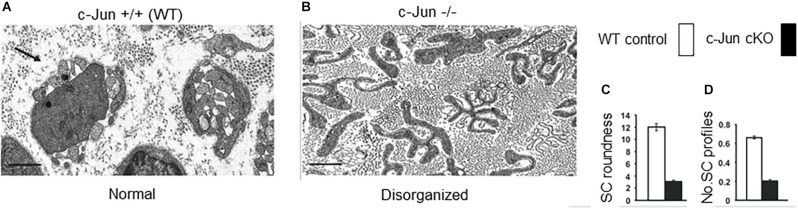
The structure of regeneration tracks (Bungner bands) is regulated by Schwann cell c-Jun. Electron micrographs showing transverse sections of the distal stump of mouse sciatic nerve 4 weeks after cut. **(A)** WT nerve showing normal Bungner bands. **(B)** c-Jun cKO nerve showing distorted Bungner bands. **(C,D)** Quantification of the roundness and number of Schwann cell profiles in Bungner bands. Scale bar 1 μm (modified from Arthur-Farraj et al., 2012).

The importance of c-Jun for the Schwann cell injury response first became clear when it was found that in mice lacking c-Jun specifically in Schwann cells (c-Jun cKO mice), sciatic or facial nerve injury results in a broad spectrum of regenerative abnormalities (Figures 5–7; Arthur-Farraj et al., 2012; Fontana et al., 2012; reviewed in Jessen and Mirsky, 2016, 2019b). In these mice, axonal injury causes excessive death of sensory and motor neurons (Figure 5). Axonal regeneration *in vivo* is strongly compromised, and in line with this cell culture studies show directly that c-Jun levels in Schwann cells control the elongation rate of the axons associated with them (Arthur-Farraj et al., 2012; Huang et al., 2019). In the c-Jun cKO mice, down-regulation of myelin genes after an injury is delayed and incomplete, activation of myelinophagy is suppressed (Gomez-Sanchez et al., 2015) and myelin clearance is compromised (Figure 6). *In vitro*, c-Jun knockout Schwann cells adopt a flattened morphology rather than the typical bi-or tri-polar shape of cultured Schwann cells, and in line with this, the regeneration tracks (Bungner bands) in the cut nerves of c-Jun cKO mice are disorganized (Figure 7). In the distal stump of cut c-Jun cKO nerves, 173 genes are differentially expressed compared to cut wild type nerves and six miRNAs are also dis-regulated. At the protein level, NCAM and p75NTR are over-expressed while NCAD is under-expressed (Arthur-Farraj et al., 2012, 2017). It is striking that this c-Jun regulated program represents a relatively small fraction of the molecular changes induced by injury which extends to some 4,000–5,000 genes. This is in line with the notion that c-Jun has an important but restricted function in injured nerves, specifically regulating the collection of events involved in the reprogramming of Schwann cells to generate repair cells. Thus c-Jun functions as an essential global amplifier of the repair cell phenotype.

## Maintaining Schwann Cell c-Jun Prevents Regeneration Failure Due to Age and Chronic Denervation

The twin observations that c-Jun boosts repair cell function and that c-Jun levels are reduced as the cells lose the capacity to support regeneration in older animals and during chronic denervation, suggest a causal link. Namely that the failure of repair cells in these two situations is a consequence of reduced c-Jun. If this were the case, preventing the loss of c-Jun should also prevent the decline in regeneration. This question was addressed in a recent publication (Wagstaff et al., 2021). To prevent the reduction in c-Jun in older or chronically denervated Schwann cells, the authors generated a mouse in which Schwann cells alone expressed a c-Jun transgene in addition to endogenous c-Jun (c-Jun OE/+ mice). In these mice, c-Jun levels in uninjured nerves were still low, although about seven-fold higher than the very low levels in wild type control mice, and the nerves were normal except for a slight reduction in myelin thickness (Fazal et al., 2017).

Studying the effects of age, Wagstaff et al. (2021) found that while the c-Jun response to acute nerve injury (3-day cut) in middle-aged wild type mice reached only ~50% of that in young ones, this drop was not seen in middle-aged c-Jun OE/+ mice, where c-Jun levels after injury remained similar to those in young ones. Measurements of regeneration after nerve crush using neuron backfilling showed that in wild type mice, age reduced regeneration by about 50%, as expected. This was not the case in c-Jun OE/+ mice, however, where regeneration capacity was undiminished by age, similar numbers of motor and DRG neurons regenerated in the nerves of young and middle-aged mice (Wagstaff et al., 2021).

Analyzing chronic denervation, it was found that one week after injury, c-Jun elevation was similar in wild type and c-Jun OE/+ mice. But in c-Jun OE/+ mice, c-Jun levels stayed constant during a subsequent 10 week period of chronic denervation, while they dropped by about 60% in wild type nerves (Wagstaff et al., 2021). Comparing regeneration through 1-week and 10-week denervated distal stumps of the c-Jun OE/+ and wild type mice showed, first, that regeneration through short-term (1-week) denervated stumps was similar in the two mouse strains, where c-Jun levels were similar; second, that in wild type mice regeneration through chronically (10-week) denervated stumps was substantially reduced, as expected, and third, that in c-Jun OE/+ mice, regeneration through 10-week denervated stumps, where c-Jun levels are maintained, did not decline but remained similar to that seen with 1-week stumps. These results were obtained both with spinal cord motor neurons and DRG sensory neurons. In these experiments, therefore, preventing the decline in Schwann cell c-Jun levels during chronic denervation prevented the decline in axonal regeneration (Wagstaff et al., 2021).

**Figure 8:**
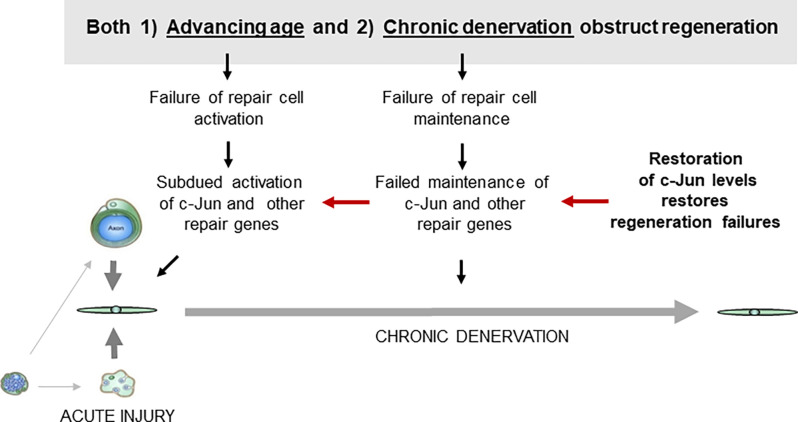
Failure of c-Jun expression in repair cells prevents successful regeneration. Defective c-Jun expression in repair cells is an important factor in regeneration failures associated with both aging and chronic denervation. Because correcting defective c-Jun levels corrects regeneration deficits, pathways that regulate c-Jun levels are a potential target for treatments for improving nerve regeneration (for references see text).

These experiments suggest that defective c-Jun expression in repair cells is an important factor in the regeneration failures, particularly those associated with both older age and chronic denervation (Figure 8). They show also that correcting defective c-Jun levels corrects regeneration deficits, pointing to the c-Jun regulated program as a target for treatments aimed at improving nerve regeneration.

## The c-Jun Regulated Schwann Cell Response to Neurological Disease

Schwann cell c-Jun is elevated in several human neuropathological conditions, although these disease states do not involve mechanical injury to axons, i.e., transection or crush, as mentioned above (Hutton et al., 2011; Wilcox et al., 2020, 2021). Increased c-Jun levels in uninjured nerves have also been studied in mice, both in disease models and c-Jun over-expressing mutants. This work has given rise to the idea that, in addition to the function of high Schwann cell c-Jun after injury, a more modest c-Jun elevation in uninjured nerves, even in Schwann cells that still retain myelin and Remak phenotypes, also activates a neuron-supportive Schwann cell program that protects against neurological damage.

This is based on studies on the dose-dependence of c-Jun function using heterozygous (c-Jun OE/+) and homozygous (c-Jun OE/OE) c-Jun over-expressing mice, and on examining mouse models of Charcot-Marie-Tooth (CMT)1A and CMT1X disease (Hantke et al., 2014; Klein et al., 2014; Fazal et al., 2017). In c-Jun homozygous over-expressing mice (c-Jun OE/OE mice; Fazal et al., 2017), a 28-fold elevation of c-Jun in Schwann cells causes hypomyelination pathology, implicating c-Jun as a candidate gene in demyelinating neuropathies. On the other hand, and as mentioned before, nerves in c-Jun heterozygous over-expressing mice (c-Jun OE/+ mice) where c-Jun is about 6-fold higher than WT are essentially normal (Fazal et al., 2017). Substantially normal myelin and Remak phenotypes despite significant elevation of c-Jun protein are also seen in mouse models of CMT1A and CMT1X (Hantke et al., 2014; Klein et al., 2014). Importantly, these CMT1 models reveal that already at low or moderate c-Jun levels compatible with myelination, c-Jun promotes neuron-supportive signaling from Schwann cells to neurons. Thus, in the C3 mouse model of CMT1A, Schwann cell c-Jun is elevated, but not sufficiently to disrupt myelination. Nevertheless, this results in a marked increase in axonal survival and sensory-motor performance in this disease model (Hantke et al., 2014). In Cx32def mice that mimic CMT1X Schwann cell c-Jun is also elevated. This does not disrupt myelin but is accompanied by increased GDNF expression in myelin Schwann cells (Klein et al., 2014). This suggests that the c-Jun elevation seen in pathological human nerves may indicate an adaptive neuron-supportive Schwann cell response to disease (Hutton et al., 2011; Wilcox et al., 2020, 2021).

While therapeutic boosting of the c-Jun pathway in diseases such as CMT1A might reduce axonal loss, it would need careful adjustment due to the potentially demyelinating effect of high c-Jun levels. Concerning the targeting of the Schwann cell c-Jun pathway for promoting regeneration after an injury it is important to note, first, that there is no evidence that elevation of c-Jun, such as that in the c-Jun overexpressing mice discussed above, is tumorigenic. Theoretically, this is, in any case, unlikely since after injury, wild type Schwann cells re-enter the cell cycle, alter their differentiation state and express very high levels of c-Jun, yet do not develop tumors. As expected, therefore, no tumors were found in 10-month-old homozygous c-Jun overexpressing mice (c-Jun OE/OE mice; Fazal et al., 2017). Second, the elevation of c-Jun that effectively promotes regeneration is compatible with myelination and functional recovery after injury. Thus, in c-Jun OE/+ mice, c-Jun overexpression is sufficient to fully correct regeneration deficits due to advancing age and chronic denervation, as discussed above (Wagstaff et al., 2021). Nevertheless, after nerve crush, sciatic nerves in these mice regenerate to achieve full functional recovery and myelination, although both proceed with a delay and myelin is somewhat thinner than WT, as seen also before injury (Fazal et al., 2017). As discussed further below, time limited and reversible elevation of c-Jun, for instance by hijacking the autocrine signaling loops of repair cells, would likely be an effective therapy for promoting nerve regeneration.

## A Comparison Between Repair Cells and Immature Schwann Cells

Because repair Schwann cells in the adult, and immature Schwann cells in developing nerves, carry out quite different functions, it is not surprising that these cells also diverge in many other ways. This includes a strikingly dissimilar structure, distinct molecular expression and lineage relationships and numerous differences in the molecular machinery that regulates them.

**Figure 9:**
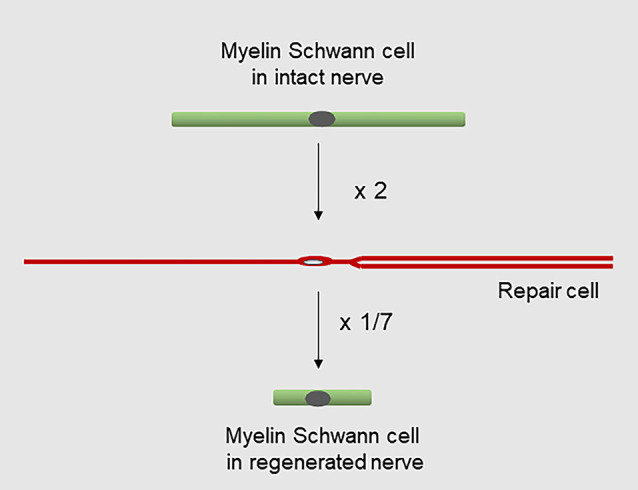
Changes in Schwann cell length during injury and regeneration. Myelin cells elongate about two-fold to generate repair cells, while repair cells shorten about seven-fold to generate new myelin cells after regeneration (for references see text).

The structure of repair cells is adapted to form the compact cellular columns (Bungner bands) that function as essential regeneration tracks for regrowing axons (Figures 4, 7). As myelin and Remak cells transform to repair cells they undergo a surprising elongation that, together with two-three fold increase in Schwann cell number, will maximize cellular overlap within Bunger bands, and therefore the formation of uninterrupted tracks. Thus, repair cells are about two and three-fold longer than myelin and Remak cells, respectively and seven- to 10-fold longer than immature Schwann cells (Figures 3, 9). Repair cells also adopt a spiraling, corkscrew-like structure and frequently branch forming long parallel processes, both of which are likely to contribute to the generation of tight cellular columns (Gomez-Sanchez et al., 2017). While immature Schwann cells derive from Schwann cell precursors in embryonic nerves (Jessen and Mirsky, 2019a), these and other lineage tracing studies also show conclusively that repair cells in injured nerves derive from adult myelin and Remak cells and revert to those phenotypes after regeneration (Gomez-Sanchez et al., 2017; Stierli et al., 2018).

It is well established that the myelin cells that form in nerves after regeneration are short, measuring only 25–30% of the length of myelin cells before injury (Jacobs and Cavanagh, 1969; Gomez-Sanchez et al., 2017). Remarkably, this means that repair cells undergo a radical shortening to only ~13% of their length to form the short internodes of regenerated nerves (Figure 9). The cytoskeletal mechanisms that control the striking elongation and shortening of Schwann cells during injury and regeneration remain to be elucidated.

The distinct structure of repair cells is in part controlled by c-Jun. The regeneration tracks that form in c-Jun cKO mice are conspicuously abnormal, loosely composed of irregular and flattened cells (Figure 7; Arthur-Farraj et al., 2012). Even in cell culture, Schwann cells from c-Jun cKO mice show increased flattening and roundness and lack of processes, instead of the characteristic bi- or tri-polar *in vitro* morphology of wild type Schwann cells, that reflects their structure *in vivo*. c-Jun is therefore an intrinsic regulator of the structure of denervated Schwann cells (Arthur-Farraj et al., 2012).

Probably the clearest example of a difference in molecular regulation between developing and repair Schwann cells is provided by c-Jun because this factor is unimportant in immature cells but is a key regulator of adult repair cells. Other examples include the tumor suppressor protein merlin, which is essential for the Schwann cell response to injury and nerve regeneration, although myelination is only slightly and transiently affected in mice with inactivation of merlin in Schwann cells (Mindos et al., 2017). Similarly, chromatin modifications, involving H3K27 demethylations and acetylation and H3K4 methylation promote up-regulation of injury genes and down-regulation of myelin genes in repair cells, although these events are not involved in controlling developmental myelination (Hung et al., 2015; Ma et al., 2016; reviewed in Ma and Svaren, 2018). The transcription factor STAT3 is also unimportant for Schwann cell development but important for long-term repair cell maintenance (Benito et al., 2017).

Several other differences in the molecular regulation between repair cells and developing Schwann cells have also been identified. Autocrine neuregulin signals promote re-myelination by repair cells but not myelination by developing Schwann cells, and axon-associated neuregulin is necessary for developmental myelination but acts only as a timer for re-myelination by repair cells (Fricker et al., 2013; Stassart et al., 2013). Further, ERK1/2 promotes myelin thickness in developing cells but not during re-myelination in the adult, and injury-induced proliferation associated with repair cell generation is controlled by cyclin D1, while this protein does not control the proliferation of developing cells (Kim et al., 2000).

Together these findings demonstrate that the generation of repair cells, and the regulation of their function, depends on mechanisms, including c-Jun, that have minor or no roles during development.

## The Pharmacology of Regeneration: Hijacking The Autocrine Loops That Support Repair Cells

There is still no generally recognized pharmacological treatment for the promotion of nerve repair. Schwann cells have multiple roles in regeneration, and repair cell malfunction underlies important regeneration failures. It is therefore an attractive option to develop treatments that promote the capacity of repair cells to support regeneration. In support of this, the work on Schwann cell c-Jun outlined above serves as a proof of principle. Namely, that manipulating signaling in repair Schwann cells can be an effective way to promote axonal regeneration.

**Figure 10:**
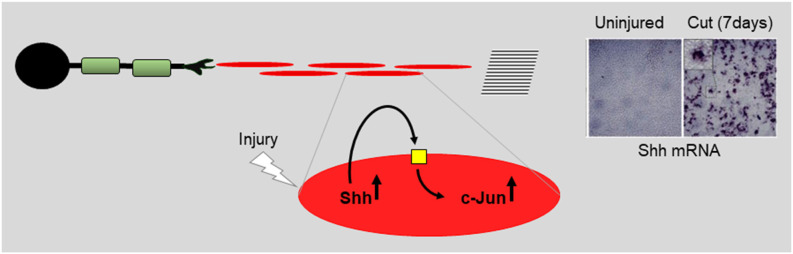
Repair cells are supported by autocrine signaling loops. Using data on Shh (Wagstaff et al., 2021) the cartoon illustrates the notion that injury activates autocrine loops that support repair cells. Application or enhanced expression of autocrine signals constitute interesting candidates for future therapeutic use.

Therefore, it is interesting that a group of Schwann cell derived signals, exemplified by Shh, neuregulin, TGFß, GDNF, and IGF1, appear to work as endogenous boosters of repair cells. These factors are up-regulated in repair cells, act through autocrine loops to promote repair cell function, and promote regeneration when applied to damaged nerves *in vivo* (Figure 10). Targeting these pathways is a promising approach for the development of tools for therapeutic use. The data implicating these factors in autocrine repair cell support is outlined below.

### Sonic Hedgehog

The most recently identified protein that appears to function in this way is Sonic hedgehog (Shh) (reviewed in Moreau and Boucher, 2020). Shh and Gli1, one of the downstream effectors of Shh signaling, are up-regulated in repair cells after injury, while Shh is also expressed in DRG neurons (Hashimoto et al., 2008; Arthur-Farraj et al., 2012; Martinez et al., 2015; Yamada et al., 2018). During chronic denervation, Schwann cell Shh levels gradually fall, like those of c-Jun. In cultured Schwann cells, Shh agonists up-regulate c-Jun protein and c-Jun phosphorylation and promote the expression of two c-Jun targets, GDNF and BDNF (Hashimoto et al., 2008; Wagstaff et al., 2021). Further, *in vivo*, genetic inactivation of Shh selectively in Schwann cells results in reduced c-Jun expression and phosphorylation after injury and reduced levels of the c-Jun target p75NTR in repair cells. Additional support for a role for endogenous Shh in nerve repair comes from the observation that administration of cyclopamine, a specific blocker of Shh signaling, to injured nerves suppressed the normal injury-induced expression of BDNF and increased the injury-induced death of motoneurons (Hashimoto et al., 2008). Conversely, after nerve injury *in vivo*, exposure to Shh or Shh agonists promotes nerve regeneration in several experimental paradigms (Pepinsky et al., 2002; Bond et al., 2013; Martinez et al., 2015; Yamada et al., 2018). Since Schwann cells are the major source of Shh in injured nerves, all of this is consistent with autocrine Shh signaling in Schwann cells after injury. This suggestion is supported by the observation that the addition of cyclopamine alone to purified Schwann cell cultures suppresses c-Jun and c-Jun phosphorylation (Wagstaff et al., 2021).

Taken together, these experiments suggest that injury-activated Shh from repair cells promotes regeneration directly by acting on neurons (Martinez et al., 2015) and indirectly by acting back on repair cells to promote the expression of c-Jun and the repair cell phenotype (Figure 10).

### Neuregulin

While axon-associated neuregulin 1 type lll is an obligatory survival signal for Schwann cell precursors (Jessen et al., 1994; Dong et al., 1995) and drives developmental myelination (Taveggia et al., 2005), soluble neuregulin 1 type l is expressed by adult Schwann cells and has a role in repair. Nerve injury triggers a strong elevation of neuregulin 1 type l in the Schwann cells of the distal stump that peaks a few days after injury but is significantly reduced after 10–14 days as axons regenerate and re-myelination takes place (Stassart et al., 2013). In chronically denervated Schwann cells, neuregulin 1 expression also declines, albeit much more slowly (Ronchi et al., 2017). Neuregulin 1 applied to regenerating nerves promotes repair in a variety of settings (Mancuso et al., 2016; Yasui et al., 2016; reviewed in Gambarotta et al., 2014; El Soury and Gambarotta, 2019). The mechanism by which this works is likely to be *via* direct effects on repair cells and perhaps macrophages. Neuregulin 1 activates multiple signaling pathways in Schwann cells including the PI3K–Akt pathway, ERK1/2–MAPK, and calcineurin–NFAT pathways among others (reviewed in Fricker and Bennett, 2011) and can promote Schwann cell proliferation and migration (Chang et al., 2013). Increased neuregulin signaling to repair cells *in vivo*, by overexpression of ErbB2 receptors strongly elevates c-Jun expression and promotes axonal regeneration (Han et al., 2017). In agreement, soluble neuregulin 1 type l applied at relatively high concentrations *in vitro*, activates c-Jun and other genes associated with the injury response and suppresses myelin genes (Syed et al., 2010; El Soury et al., 2018). These effects are concentration-dependent, since at lower concentrations, soluble neuregulin 1 promotes myelination (Syed et al., 2010). The effects of neuregulin 1 are also context-dependent and regulated by the cAMP pathway, an important driver of myelination (Monk et al., 2015). When cAMP activation is weak, a high concentration of soluble neuregulin 1 drives Schwann-cell proliferation (Arthur-Farraj et al., 2011), and under these conditions neuregulin 1 induces Schwann cells to secrete factors that promote sympathetic neuron survival and axon outgrowth (Mahanthappa et al., 1996), features characteristic of the repair cell phenotype. However, when cAMP activation is strong, neuregulin 1 triggers the appearance of non-dividing cells that express myelin genes (Arthur-Farraj et al., 2011).

These data are consistent with the view that Schwann cell neuregulin 1 activation functions in an autocrine manner to support nerve repair. Early after injury, high levels of Schwann cell derived neuregulin suppress myelin genes, activate c-Jun and promote Schwann cell reprogramming to repair cells resulting, indirectly, in the stimulation of axonal regeneration (Mahanthappa et al., 1996; Gambarotta et al., 2014). At later times, when myelination starts, neuregulin 1 levels are lower and cAMP signaling stronger, and neuregulin 1 promotes myelination (Syed et al., 2010; Stassart et al., 2013).

### Glial Derived Neurotrophic Factor (GDNF)

GDNF is expressed in Schwann cells and up-regulated at the mRNA and protein level after injury, while during chronic denervation, GDNF levels, like those of Shh and c-Jun, gradually decline (Trupp et al., 1995; Naveilhan et al., 1997; Höke et al., 2000, 2002; Barras et al., 2002; Eggers et al., 2010; Fontana et al., 2012; Xu et al., 2013). GDNF is a survival factor for spinal cord motor neurons and a subpopulation of DRG neurons, and application of GDNF to injured adult peripheral nerves protects the spinal cord and facial motor neurons and promotes axonal regeneration (Li et al., 1995; Yan et al., 1995; Naveilhan et al., 1997; Bennett et al., 1998; Chen et al., 2001; Piquilloud et al., 2007; Eggers et al., 2008). GDNF acts on Schwann cells through GFRalpha1/NCAM signaling to affect multiple intracellular pathways, including ERK1/2, CREB, PKA, and PKC (Iwase et al., 2005). Notably, enhanced expression of GDNF in Schwann cells promotes the repair Schwann cell state and inhibits the switch to myelination (Eggers et al., 2013). GDNF is therefore likely to promote regeneration directly and indirectly through action on repair cells and neurons.

### Transforming Growth Factor ß (TGFß)

TGFß Is expressed and secreted from Schwann cells and up-regulated after nerve injury (Unsicker et al., 1991; Scherer et al., 1993; Einheber et al., 1995; Stewart et al., 1995; Li et al., 2015). TGFß has multiple effects on Schwann cells. TGFß induces Schwann cell migration (Clements et al., 2017), and promotes either proliferation or apoptosis depending on the status of several other parameters. These include c-Jun levels, cell differentiation state, cell density, and the presence of other signals such as neuregulin 1 or activation of cAMP pathways (D’Antonio et al., 2006; Li et al., 2015 and references therein). TGFß inhibits cAMP-dependent myelin protein expression in Schwann cell cultures and myelination in neuron-Schwann cell cocultures (Morgan et al., 1994; Einheber et al., 1995; Stewart et al., 1995). TGFß applied to cultured Schwann cells also activates c-Jun (Parkinson et al., 2001), and up-regulates the adhesion molecules NCAM and L1 that are expressed by repair cells, but not myelin cells. All of this, suggests that the autocrine effect of TGFß after nerve injury is to promote Schwann cell reprogramming and the expression and function of the repair cell phenotype. Importantly, there is also evidence that TGFß is required for the neurotrophic effect of GDNF, a factor that, in turn, is thought to be central for nerve repair (see above; reviewed in Krieglstein et al., 2002). Potentially, TGFß also suppresses the function of macrophages in injured nerves, a function that has been suggested to promote nerve regeneration (Vidal et al., 2013).

Application of TGFß to injured peripheral nerves is reported to promote regeneration although this field has not yet been studied extensively (Sulaiman and Dreesen, 2014; Wang et al., 2016; reviewed in Sulaiman and Nguyen, 2016; Li et al., 2017). The underlying mechanisms are unclear. In view of the multiple functions of TGFß, they are likely to be complex and include the promotion of Schwann cell reprogramming and expression of repair supportive features by these cells, promotion of GDNF signaling to neurons, as well as immunosuppression, in addition to direct effects on regenerating axons.

### Insulin-Like Growth Factor 1 (IGF-1)

IGF1, IGF binding proteins, and the type 1 IGF receptor are up-regulated in Schwann cells of injured nerves (Pu et al., 1995; Cheng et al., 1996; Hammarberg et al., 1998; reviewed in Sullivan et al., 2008). IGF-1 regulates several functions in Schwann cells that are relevant to the essential activity of repair cells after injury. This includes the promotion of cell motility, process extension, survival, and proliferation (Stewart et al., 1996; Syroid et al., 1999; Cheng et al., 2000; Delaney et al., 2001). Potentially, IGF-1 also promotes myelination, since in a defined culture medium in the presence of low cAMP pathway activation, IGF-1 induces the expression of the myelin lipid galactocerebroside and the major myelin protein P0 (MPZ; Stewart et al., 1996). IGF-1 also acts directly on neurons to support neurite outgrowth *in vitro*, and *in vivo* application of IGF-1 to injured nerves promotes regeneration in several different settings (Nachemson et al., 1990; Fansa et al., 2002; Apel et al., 2010; Bayrak et al., 2017; reviewed in Sullivan et al., 2008; Slavin et al., 2021). This is likely due to the combination of autocrine IGF-1 action to promote repair cell function and direct effects on neurons.

In addition to the molecules reviewed above, other factors, including neurotrophins, FGF2, PDGF, and VEGF may also function to support repair cells, in addition to their other effects in injured nerves, although the evidence, outlined below, is less complete.

### Neurotrophins

These factors have been extensively studied in the context of Schwann cell development and myelination, but the role of neurotrophins in these processes is still unclear (reviewed in Xiao et al., 2009). Although Schwann cells express the neurotrophin receptors p75NTR, TrkC, and truncated TrkB, and Schwann cell levels of NGF, NT4, and BDNF increase after injury, it is unclear to what extent this represents autocrine signaling loops. The expression of Trk receptors on neurons is widespread and the application of neurotrophins to injured nerves increases axon sprouts. After chronic denervation when BDNF levels in repair cells have declined, application of BDNF increases the number of regenerating neurons, but in most other experimental situations it remains to be resolved whether application of neurotrophins to injured nerves promotes significant axonal regeneration, or is mostly restricted to inducing axonal sprouting (reviewed in Gordon, 2009).

### Fibroblast Growth Factor 2 (FGF2)

FGF2 and FGF receptors (FGFR) are up-regulated in Schwann cells and neurons after nerve injury. Yet, much remains to be learned about the role of this system in nerve repair, since the effects of FGF2 appear to be subtle and complex and depend on the neuron type, FGF2 isoform, and FGFR type involved (reviewed in Grothe and Nikkhah, 2001; Grothe et al., 2006). *In vitro*, FGF2 stimulates Schwann cell proliferation and inhibits myelin gene expression (Davis and Stroobant, 1990; Morgan et al., 1994), and studies on mice in which FGF2 is deleted or over-expressed suggest that these effects can also be seen near the site of nerve crush *in vivo* (Haastert et al., 2006; reviewed in Grothe et al., 2006). DRG neurons also up-regulate FGF2 and FGFRs after injury and FGF2 appears to act directly on neurons to stimulate neurite outgrowth (Unsicker et al., 1987 and references therein; Fujimoto et al., 1997). There is evidence that applied FGF2 promotes axonal regeneration across regeneration inserts and accelerates the functional recovery of sensory fibers (Aebischer et al., 1989; Haastert et al., 2006), but it is unclear to what extent these or other effects of FGF2 *in vivo* are due to direct effects on Schwann cells. Further, after nerve injury, up-regulation of FGF2 and FGFR is restricted to the site of damage (Grothe et al., 2001), suggesting a limited role for this system in the repair cells of the distal stump.

### Platelet-Derived Growth Factor (PDGF)

PDGF and PDGF receptors are up-regulated in Schwann cells after nerve injury (Oya et al., 2002; Yamazaki et al., 2009), and cultured Schwann cells secrete PDGF that promotes both Schwann cell proliferation and survival (Davis and Stroobant, 1990; Eccleston et al., 1990; Hardy et al., 1992; Watabe et al., 1994; Meier et al., 1999; Lobsiger et al., 2000). Neurons also express PDGF receptors and it is likely that PDGF acts directly on neurons to promote neurite extension (Smits et al., 1991; Eccleston et al., 1993; Nakao et al., 1995). There is evidence that applied PDGF promotes nerve regeneration *in vivo*, although this appears not to have been studied extensively (Golzadeh and Mohammadi, 2016; Hong et al., 2018; see however Welch et al., 1997). These data suggest that in injured nerves, PDGF works through an autocrine PDGF loop to support survival and proliferation of repair Schwann cells, in addition to PDGF acting directly on regenerating axons.

### Vascular Endothelial Growth Factor (VEGF)

VEGF is expressed by Schwann cells in uninjured nerves and in culture, but does not appear to be up-regulated by Schwann cells after nerve injury (Taiana et al., 2014; Muratori et al., 2018). There is evidence that VEGF promotes Schwan cell migration (Sondell et al., 1999a; Muratori et al., 2018), but stimulation of proliferation is controversial (Sondell et al., 1999b; Muratori et al., 2018). Applied VEGF promotes regeneration after nerve injury (Pereira Lopes et al., 2011; Wu et al., 2021), which might be due to enhanced vascularization (Sondell et al., 1999a) or direct effects on neurons (reviewed in Rosenstein et al., 2010). It has also been proposed that Schwann cell derived VEGF acts through an autocrine loop to support Schwann cells (Rosenstein et al., 2010).

## Conclusions

The presence of functional repair cells is indispensable for effective regeneration. It is therefore unfortunate that the expression of their phenotype is not robust: repair cell activation falters as animals get older and the repair phenotype fades during chronic denervation. These malfunctions are important reasons for the poor outcomes after nerve damage in humans. It is essential, therefore, to learn about the signals that control and maintain repair cells. In this context, the transcription factor c-Jun is important for several reasons: First, because it acts as an amplifier of the repair phenotype and is required for the generation of functional repair cells after injury. Second, failure of c-Jun expression is linked to failures of axonal regeneration in older animals and during chronic denervation. Third, manipulation of c-Jun signaling in repair cells has proved an effective way of promoting axonal regeneration. This demonstrates, in principle, that targeting repair cells to boost their function is an efficient way of improving nerve repair.

One way to amplify the function of repair cells would be to hijack endogenous autocrine support mechanisms since there is increasing evidence that repair cells are sustained by several autocrine signaling loops. The development of methods for the localized and timed delivery of such signals to injured nerves is an attractive option for the clinical improvement of nerve injuries.

## Author Contributions

Both authors contributed equally to the writing and editing of the manuscript. All authors contributed to the article and approved the submitted version.

## Conflict of Interest

The authors declare that the research was conducted in the absence of any commercial or financial relationships that could be construed as a potential conflict of interest.

## Publisher’s Note

All claims expressed in this article are solely those of the authors and do not necessarily represent those of their affiliated organizations, or those of the publisher, the editors and the reviewers. Any product that may be evaluated in this article, or claim that may be made by its manufacturer, is not guaranteed or endorsed by the publisher.
